# Hepatitis C Vaccines, Antibodies, and T Cells

**DOI:** 10.3389/fimmu.2018.01480

**Published:** 2018-06-28

**Authors:** Naglaa H. Shoukry

**Affiliations:** ^1^Centre de Recherche du Centre hospitalier de l’Université de Montréal (CRCHUM), Montréal, QC, Canada; ^2^Département de médecine, Faculté de médecine, Université de Montréal, Montréal, QC, Canada

**Keywords:** hepatitis C, vaccines, antibodies, T cells, reinfection

## Abstract

The development of vaccines that protect against persistent hepatitis C virus (HCV) infection remain a public health priority. The broad use of highly effective direct-acting antivirals (DAAs) is unlikely to achieve HCV elimination without vaccines that can limit viral transmission. Two vaccines targeting either the antibody or the T cell response are currently in preclinical or clinical trials. Next-generation vaccines will likely involve a combination of these two strategies. This review summarizes the state of knowledge about the immune protective role of HCV-specific antibodies and T cells and the current vaccine strategies. In addition, it discusses the potential efficacy of vaccination in DAA-cured individuals. Finally, it summarizes the challenges to vaccine development and the collaborative efforts required to overcome them.

## Introduction

Direct-acting antivirals (DAAs) against hepatitis C virus (HCV) infection can achieve complete cure in >95% of cases (1, 2) and have been suggested to have the potential to eliminate an infection that affects more than 71 million individuals worldwide (3). It was suggested that with such effective treatments, a vaccine against HCV may no longer be needed (4). However, this argument may be overly optimistic. HCV infection can remain asymptomatic for years during which many infections go undiagnosed. It is estimated that only 5% of HCV cases worldwide are diagnosed (5, 6). Many current and new HCV infections occur in developing countries and among marginalized populations like people who inject drugs (PWIDs), incarcerated individuals, and men who have sex with men (7). These individuals are mostly disengaged from medical care with limited access to HCV screening and treatment. In the meantime, they continue to infect others and contribute to the ongoing epidemic. Indeed, the current opioid epidemic in North America has been associated with increased incidence of HCV (8). Medical procedures remain the major cause of new HCV infections in developing countries with high prevalence of HCV posing another risk factor within the general population and travelers to these areas (9). Finally, DAA treatment does not protect against reinfection further underscoring the need for an effective vaccine (10).

The World Health Organization (WHO) has set elimination targets for 2030 to reduce the rate of new HCV infections by 90% (11). Despite numerous success stories for implementation of national hepatitis C strategies with increased screening, diagnosis, and treatment, notably in places like the Republic of Georgia and Egypt (12–15), this will not be enough to curb HCV transmission on the long-term. Successful vaccination strategies at the population level have been the only reliable method to limit transmission of different viral infections by providing herd immunity (16), especially among vulnerable populations and in low-resource settings. The ongoing effort to eliminate HCV should include two arms: screening and treatment, and enhanced prevention *via* vaccination and harm reduction measures.

The quest to develop a vaccine against HCV has been active, since the discovery of the virus in 1989 but it has been a challenging endeavor due to the high variability of the virus and the lack of small animal models for preclinical testing. Strategies have aimed at either producing broadly neutralizing antibodies (bNAbs) that would neutralize the infectivity of the virus or generating potent virus-specific CD4 and CD8 T cells that can eliminate infected hepatocytes. Various adjuvants, vectors, and vaccination regimens have been tested over the years. At present, two vaccines have made it into human preclinical and clinical trials. The first is a recombinant form of the virus envelope glycoproteins gpE1 and gpE2 aimed at inducing neutralizing antibodies and CD4 helper T cells (17, 18). The second is a vector-based vaccine encoding nonstructural (NS) proteins of the virus (NS3-NS5) using chimpanzee adenovirus priming and modified vaccinia Ankara (MVA) boost. This vaccine regimen was shown to induce high frequencies of virus-specific polyfunctional CD4 and CD8 T cells in healthy volunteers (19) and is currently in phase 2 clinical trials in PWIDs (NCT01436357). Results of this clinical trial are pending and will inform the field about the most appropriate future direction to follow.

This article reviews what we know about the role of antibodies versus T cells in mediating protective immunity against HCV and the pros and cons of targeting each approach in vaccine development. It also discusses the current challenges to HCV vaccine research and suggested collaborative efforts to overcome them.

## Correlates of Immune Protection During Acute HCV

Approximately 25% of individuals acutely infected with HCV are able to eliminate the virus spontaneously while the rest develop persistent infection and chronic liver disease, including fibrosis, cirrhosis, and hepatocellular carcinoma (20). The successful development of a vaccine against HCV is essentially informed by correlates of protective immunity induced during acute resolving infection. Multiple studies in humans and chimpanzees have clearly demonstrated the kinetic association of spontaneous viral clearance with induction of a broad, sustained HCV-specific CD4 and CD8 T cells [reviewed in Ref. (21)]. These T cell responses were also polyfunctional, producing multiple cytokines and effector functions (22). As the infection is cleared, virus-specific T cells develop a memory T cell phenotype and upregulate cell surface expression of the IL-7 receptor CD127 (22–25). The majority of HCV-infected individuals do generate a relatively broad CD4 and CD8 T cell response early on after infection that may afford partial control of viremia. Nevertheless, the abrupt loss of CD4 helper T cell responses, compromises CD8 T cell functionality and facilitates emergence of viral escape mutations in targeted CD8 T cell epitopes (26–28). As CD4 T cell functions are lost, the frequency of virus-specific T cells is reduced and the response becomes limited in breadth and/or functionality [reviewed in Ref. (21)]. Altogether, these dysfunctions result in virus rebound and persistent viremia. As the virus persists, CD8 T cells recognizing intact epitopes (i.e., epitopes that have not mutated) become exhausted and express exhaustion markers like programmed death 1 (PD1), T-cell immunoglobulin and mucin domain-containing-3 (Tim-3), cytotoxic T-lymphocyte protein 4 (CTLA4), 2B4, CD160, KLRG1, T-cell immunoreceptor with Ig and ITIM domains (TIGIT), and CD39 (21, 29). In addition, they upregulate expression of the transcription factor eomesodermin (Eomes), and in some cases the T cell factor 1 (TCF1) while downregulating expression of the T-box transcription factor (T-bet) (30, 31). T cell exhaustion leads to progressive loss of effector functions resulting in reduced polyfunctionality, cytotoxicity, and loss of proliferative capacity [reviewed in Ref. (21)]. As the infection persists, the frequencies of HCV-specific CD8 and CD4 T cells detectable in peripheral blood are dramatically reduced (32, 33). During chronic infection, HCV-specific CD8 T cells are more readily detectable in the liver albeit with an exhausted phenotype (34, 35). Interestingly, CD8 T cells targeting epitopes that have mutated remain functional and detectable in peripheral blood and acquire a CD127+ memory T cell phenotype, similar to memory T cells generated following spontaneous resolution (36).

Early studies of antibodies (Abs) against the HCV glycoproteins gpE1 and gpE2 have suggested that these responses are delayed during acute infection and are not associated with control of viremia (37). Similarly, the early use of viral pseudoparticles suggested that the development of neutralizing Abs (NAbs) is also delayed (38, 39). Yet, changes within the hypervariable region 1 (HVR1) of the gpE2 protein were detected in individuals developing chronic infection and were temporally correlated with Ab seroconversion suggesting immune selection pressure (40, 41). Furthermore, preincubation of virus inoculum with immune serum or anti-HVR1 Abs resulted in reduced infectivity in chimpanzees (42, 43). Passive immunization prolonged the incubation period in infected chimpanzees (44) and provided sterilizing immunity (45) or reduced viral loads in mouse models of HCV infection (46). Studies using multiple viral pseudoparticles representative of the variability of the virus gpE1/gpE2 region demonstrated a correlation between the generation of NAbs and spontaneous resolution (47, 48). The isolation of bNAbs from chronically infected and spontaneously resolved individuals and the capacity of these antibodies to block infectivity in mouse models of HCV infection further underscored the important protective effect of the antibody response (49–51).

## Evidence of Protective Immunity Against HCV Upon Re-Exposure and Reinfection

Spontaneous clearance of HCV infection in chimpanzees and humans generates long-lived memory T cells that can theoretically protect against reinfection (52, 53). Long-term follow-up of a cohort of German women who were accidently infected with HCV *via* a contaminated blood product demonstrated that spontaneous resolution of acute HCV generated long-lived memory T cells that can be detected up to 20 years post resolution while antibody responses waned with time (52). This observation suggested that T cell responses may provide more durable protective immunity than antibodies. Data from cohorts of PWIDs have reported reduced rates of reinfection and/or chronicity among individuals with prior immunity to HCV as compared to HCV naïve controls with the same risk exposure (54). Studies in humans and chimpanzees have demonstrated that subsequent HCV exposures in individuals with resolved infection result in lower viral loads and shorter viremia as compared to primary infection in the same individuals (53, 55–60). HCV rechallenge was associated with rapid anamnestic immune response and associated with a blunted secondary infection or faster viral clearance (53, 55–57, 59). Experimental depletion of CD8 T cells from chimpanzees who had resolved primary HCV infection followed by a homologous rechallenge resulted in prolonged infection that resolved only upon recovery of virus-specific CD8 T cells (53). In a complementary experiment where CD4 T cells were depleted, the animals were never able to clear HCV reinfection and accumulated escape mutations in epitopes targeted by the CD8 response leading to viral breakthrough and persistent infection (61). These two studies underscored the important protective role of both CD4 and CD8 memory T cells in preventing HCV persistence.

Protection against viral persistence upon reinfection in PWIDs was associated with an increase in the magnitude and breadth of HCV-specific T cell responses, and polyfunctional memory T cells that can produce more than one cytokine or effector function (57, 59). Analysis of the T cell repertoire demonstrated that CD8 T cells expanding upon reinfection were derived from the memory T-cell repertoire with almost no contribution of *de novo* T cell responses. Furthermore, the T cell repertoire became more focused upon reinfection with selection of T cells of the highest functional avidity (62).

Protection from viral persistence upon reinfection was also associated with generation of cross-reactive NAbs (57). These findings re-emphasized the important role of NAbs in mediating protective immunity and as a key component of a successful vaccine against HCV.

Protective immunity upon re-exposure was not absolute as some chimpanzees and humans could still develop chronic infection despite having strong immune responses upon heterologous rechallenge or infection with variant viruses that are not recognized by the pre-existing memory T cells (59, 63). These observations underscore the importance of inducing broad memory immune responses that target multiple epitopes or variant viruses.

In conclusion, reinfection in humans and chimpanzees confirmed the importance of both T cell responses and antibodies in long-term protective immunity. Furthermore, they underscored the importance of inducing a broadly reactive T cell and antibody response to counteract the variability in the viral quasispecies in real-world exposures. The recent development of better tools to examine the humoral response against HCV like pseudoparticles that are representative of the diverse viral populations (48), isolation of broadly neutralizing antibodies (49, 50), resolution of the crystal structure of the virus E2 glycoprotein (64, 65), understanding the interactions between NAbs and gpE2 (66, 67), and development of gpE2 tetramers that allow direct visualization, characterization, and isolation of HCV-specific B cells (68) will have an important impact on our understanding of the protective role of NAbs against HCV and the design of better vaccines.

## Sterilizing Immunity vs Prevention of Persistence

The ideal goal of most vaccines is to provide sterilizing immunity that will protect against any infection upon exposure to the pathogen. This can only be achieved by induction of strong NAb responses that would neutralize infectivity. This approach has been very effective when targeting conserved viral surface proteins as is the case in vaccines against hepatitis A, B, and yellow fever. In the context of HCV, sterilizing immunity was not observed in chimpanzee rechallenge or human reinfection studies. Vaccination strategies aiming at inducing high titer bNAbs may indeed achieve sterilizing immunity but this will take time. Hence, it is likely that the first-generation vaccines will focus on achieving milder or blunted infections with lower viral loads and shorter periods of viremia and enhanced rate of viral clearance thus preventing viral persistence and protecting against chronic liver disease.

## Current HCV Vaccine Strategies

Two main vaccine strategies are currently moving forward with human trials targeting either the cellular or humoral immune response (Table 1). The first vaccine is aimed at priming HCV-specific CD4 and CD8 T cells, using an adenovirus-based vector approach and focusing on the virus NS (NS3-NS4A-NS4B-NS5A-NS5B) proteins. The first proof-of-concept for this vaccine was demonstrated in the chimpanzee model of HCV infection. Adenoviral vectors serotype 6 (Ad6) and 24 (Ad24) carrying genes coding for the HCV NS proteins (genotype 1b) were used as prime followed by a plasmid DNA boost. Chimpanzees were then challenged with a heterologous virus (H77 and genotype 1a). This regimen led to priming of cross-reactive HCV-specific CD8 T cells in blood and liver that expanded upon rechallenge and led to suppression of acute viremia and lower viral loads in 4/5 vaccinated chimpanzees as compared to unvaccinated controls where 2/5 chimpanzees developed chronic infection (69). Subsequent testing in healthy human volunteers using human Ad6 priming and chimpanzee adenovirus 3 (ChAd3) boost, primed broad, polyfunctional, and cross-reactive HCV-specific CD4 and CD8 T cells that were sustained for at least a year after boosting with the ChAd3 and exhibited the phenotypic and functional characteristics of long-lived central and effector memory T cells (70, 71). The next-generation of this vaccine involved a heterologous prime-boost vaccination strategy based on ChAd3 priming then boosting with an MVA vector. This latest regimen was tested in healthy human volunteers and demonstrated optimal priming and boosting with the generation of high frequencies of polyfunctional, broad HCV-specific memory CD4 and CD8 T cells (19). This regimen is currently in phase 2 clinical trials as a prophylactic vaccine in high-risk PWIDs (NCT01436357). Results from this trial will provide the first proof of efficacy of this vaccine in real-life exposure to HCV.

**Table 1:** Current hepatitis C virus vaccine development strategies.

Main Target	Stage	Immunogen	Vaccine regimen	Induced immune response	Potential improvements
T cells	Phase 2	NS3–NS5	Chimpanzee adenovirus 3 priming + modified vaccinia Ankara boost	Polyfunctional CD4 and CD8 T cells No antibodies (Abs)	More potent vectors (e.g., CMV) Invariant chain combination (enhanced Ag presentation) Combination with recombinant proteins Combination with immune check point blockade (for direct-acting antiviral-treated subjects)
Antibodies	Phase 1	gpE1/gpE2	Recombinant gpE1/gpE2 + adjuvant (MF59C.1)	Some CD4 T cells Broadly neutralizing antibodies	Better adjuvants Better CD8 T cell response inducers Combination with nonstructural proteins

The second vaccine is based on recombinant HCV gpE1/gpE2. This vaccine was one of the earliest vaccines tested in chimpanzees. Recombinant genotype 1a gpE1/gpE2 vaccination demonstrated effective immunogenicity and protective immunity against homologous or heterologous HCV rechallenge and even sterilizing immunity in some animals (72, 73). Preclinical evaluation of gpE1/gpE2 adjuvanted with MF59C.1 (an oil-in-water emulsion) in human volunteers induced NAbs as well as proliferative CD4 T cell responses against gpE1/gpE2 (17, 74). This NAb response was cross-reactive and targeted multiple epitopes (18, 75).

These two vaccines targeting either T cell responses or antibodies have demonstrated considerable immunogenicity in healthy volunteers and chimpanzees but whether they will provide protection during real-life exposures remains to be determined. Pending results of ongoing clinical trials will inform the future strategies of vaccine development. Next-generation vaccines against HCV will likely combine both T cell and antibody-based approaches into one single vaccine. The use of other strategies that may enhance immunogenicity like cytomegalovirus-based vectors (76) or fusion of the encoded antigen to major histocompatibility complex class II-associated invariant chain (Ii) (77) that have been reported to enhance CD8 T cell responses can be considered. The development of novel adjuvants and/or strategies that would tailor the T cell and antibody repertoires including the optimal vaccine/boost regimens and schedules are currently active areas of investigation.

## Vaccination in HCV-Cured Individuals

HCV reinfection following DAA-mediated viral clearance remains a problem among individuals with high-risk behaviors like PWIDs (10, 78). Hence, they are one of the main groups in need of effective vaccination strategies that will either provide sterilizing immunity or protection against viral persistence upon reinfection. DAA cure of chronic HCV can normalize the majority of innate immune responses following viral clearance (79). However, data from the few studies that examined reconstitution of HCV-specific T cell responses post DAA cure have suggested only partial restoration of virus-specific immunity. Rapid restoration of the *in vitro* proliferative capacity of HCV-specific CD8 T cells and a slight reduction in the *ex vivo* expression of PD1 on HCV-specific CD8 T cells were reported (80, 81). TCF1^+^CD127^+^PD1^+^ HCV-specific CD8 T cells expressing both exhaustion and memory markers were described in chronically infected subjects and maintained during and after treatment (82). However, these “memory-like” CD8 T cells were different as compared to conventional memory T cells as they expressed higher levels of Eomes and TCF1 and produced lower levels of IFN-γ and TNF-α upon antigenic stimulation (82). Effect of DAA treatment on restoration of HCV-specific CD4 T cells, the hallmark of protective immunity, is unknown. Whether such partial restoration of HCV-specific immunity will protect against reinfection has not been tested in humans but a preliminary study in one chimpanzee treated with DAA reported that an intrahepatic HCV-specific CD8 T cell response was maintained at 2 years following cure but was narrowly focused and failed to prevent persistence upon re-challenge (83).

Given that the HCV-specific immune response had already failed once and is likely to be incompletely restored in DAA-cured individuals, it is not clear if they will respond to vaccination and whether they will be able to generate *de novo* T cell and/or Ab responses that can mediate protective immunity. A combined DAA therapy and immunization strategy with genetic vaccines encoding the NS proteins in chimpanzees chronically infected with HCV primed multifunctional T cell responses against non-conserved MHC class I epitopes (84). However, this response failed to contain the infection with the emergence of DAA resistance mutations (84). Vaccination with the NS genes using the ChAd3 prime/MVA, that has demonstrated high immunogenicity in healthy volunteers, did not efficiently reconstitute HCV-specific T-cell immunity in HCV chronically infected patients (85). Vaccine-induced HCV-specific CD8 T-cell responses were induced in 8/12 patients but CD4 T-cell responses were rarely induced. This was true even in patients with low viral loads suppressed with interferon/ribavirin therapy. Furthermore, the overall magnitude of HCV-specific T-cells was much lower than that observed in vaccinated healthy volunteers and the HCV-specific cells exhibited a partially functional phenotype. *In vitro* expansion studies demonstrated that these specificities were derived from pre-existing low-level memory T cell populations that could be expanded by vaccination. Nevertheless, new T cell responses were induced when there was a sequence mismatch between the autologous virus and the vaccine immunogen (85). These preliminary studies suggest that it may be possible to prime new immune responses in chronic and HCV-cured individuals but it is not yet clear if they will be enough to mediate protective immunity upon re-exposure. As suggested elsewhere, it is possible that the vaccination requirements or threshold of priming a protective immune response will be different in this population (86). Additional strategies that may enhance immunogenicity like the use of novel adjuvants, vectors, or combination with immune checkpoint inhibitors may be interesting avenues to follow. Inclusion of DAA-cured individuals in upcoming vaccine trials will be necessary to evaluate immunogenicity in that special population.

## Challenges of Vaccine Development Against HCV

Several challenges remain before achieving a vaccine that can be administered to the general population. Overcoming them will require collaborative efforts from the HCV community. These challenges include.

### Virus Variability

There are 7 HCV genotypes and 67 subtypes (87). Furthermore, the virus circulates as quasispecies. Vaccination strategies targeting cross-reactive antibodies and T cell responses as well as conserved epitopes are likely to lead to better results. Data so far, suggest that the two HCV vaccines currently in progress can produce such responses, but additional methods to enhance their immunogenicity and broaden the response are needed to provide better vaccine coverage. It is also important to explore additional antigen design approaches that may overcome variability of HCV through the use of consensus sequences, ancestral, or mosaic sequences.

### Immunological Challenges

The heterogeneity in the viral populations is compounded by the diversity of the vaccine-targeted human population. Specifically, for T cell-based vaccines, it will be important to design antigens that can be presented by multiple MHC alleles. It will also be critical for vaccination regimens to overcome the intrinsic host factors associated with several of the HCV at-risk populations that may influence the immune response, including: ethnicity, age, liver disease stage, opioid usage, and HIV co-infection. Designing prime/boost strategies and vaccination schedules that would promote expansion of the ideal T cell response (eg., polyfunctional, rapid proliferation, etc.) as well as bNAbs should be considered. Furthermore, as discussed above, tailoring specific vaccination regimens that would broaden the immune response in HCV-cured individuals may be essential.

Despite the progress in our understanding of correlates of protective immunity against HCV, several basic questions remain and require additional research to decipher them. First, although we know a lot about what constitute a good immune response against HCV, we do not understand why only ~25% of infected individuals are able to generate such a response. Second, CD4 T cell dysfunction is definitely key to developing persistent viremia but mechanisms of failure/exhaustion of these CD4 T cells remain unknown. Similarly, the molecular mechanisms underlying CD8 T cell exhaustion and dysfunction remain poorly understood. Third, the function of B cells during acute and chronic HCV, the interaction between T and B cells and how this impacts development of bNAbs are understudied. Future research to answer these questions should inform the vaccine development efforts.

### Lack of Preclinical Small Animal Models

Humans and chimpanzees are the only two species that are susceptible to HCV infection. The efficacy of the two vaccines currently in clinical trials was demonstrated first in challenge studies in chimpanzees. With the moratorium on chimpanzee research, use of this model is no longer feasible (88). Furthermore, most mouse models that recapitulate the HCV life cycle are generated on immune-deficient backgrounds thus rendering them of limited use for preclinical vaccine testing but helpful in validating the *in vivo* neutralization capacity of antibodies (89). Novel mouse models using a hepacivirus isolated from the Norway rats of New York city and termed the Norway rat hepacivirus (NrHV) or rodent hepacivirus-nr-1 can recapitulate some but not all aspects of the immune response to a hepacivirus like HCV (90, 91). Specifically, depletion of CD4 T cells is required to achieve persistent viremia in this model (90). Nevertheless, it can still provide important clues about the correlates of immune protection and can be very useful in preclinical testing of novel adjuvants and vectors as well as vaccination regimen. Interestingly, NrHV infection in its original host, the rat, mimics to a great extent HCV infection in humans including the propensity to persist (92). From that perspective, the rat model of NrHV infection may be the more appropriate model to study immunity and vaccination, although the availability of rat-specific immune reagents is limited. Additional research aimed at establishing a more straight forward immune competent small animal model is still needed.

### Cohorts for Clinical Trials

One of the difficulties in clinical trials is recruitment of high risk study subjects. Given that the main risk group for HCV infection is PWIDs who also suffer from multiple social, psychological, and marginalization issues, working with this group is challenging (93). Collaborative efforts with organized cohorts around the world are ongoing (94) and should be maximized in preparation for expanding the current clinical trials or new ones. With the current availability of highly effective DAAs, it is tempting to propose clinical trials of vaccines in healthy volunteers followed by challenge and close monitoring where DAA treatment can be administered at the first sign of viral persistence. This is not a novel approach and has been used in trials for malaria vaccines (95). Evidently, the ethical implications are not trivial and will have to be considered carefully.

### Standardized Reagents, Reagent Repositories, and Immunological Methods to Assess Vaccine Efficacy

The advancement of our knowledge of protective immunity requires the availability of standardized reagents for testing as well as immune monitoring protocols. Peptides and a number of other reagents are available through the Biodefense and Emerging Infections Research Resources Repository (BEI Resources). Efforts to establish a repository for HCV pseudoparticles were discussed at the 24th International Symposium on HCV and Related Virus. Additional efforts to standardize immune monitoring among different trials should be considered.

### Funding

Hepatitis C virus research is far from over. As discussed elsewhere (96), the road to HCV elimination requires additional investment in basic research on HCV to understand the molecular mechanisms of immune control of the virus and to develop effective vaccines. In addition, large-scale clinical trials and infrastructure support for production of large-scale vaccine lots under GMP conditions are costly. Such funding will have to be secured from collaborative initiatives by governments and funding agencies across the world, the WHO, and academic-industrial partnerships.

## Concluding Remarks

Tremendous progress in the development of vaccines against HCV has occurred in recent years. The next-generation of HCV vaccines will have to target both antibodies and T cells for effective protective immunity. Better understanding of correlates of protection from viral persistence in real-life exposure settings and in DAA-cured individuals will be necessary for the design of new clinical trials. Similarly, better understanding of the humoral immune response and factors that may enhance the generation of bNAbs are warranted. Development of novel adjuvants, vectors, and vaccine prime/boost regimens that broaden the specificity and enhance the immunogenicity of vaccines are needed. Collaborative efforts for the establishment of cohorts and conducting vaccine clinical trials are essential.

## Author Contributions

NHS reviewed the literature and wrote the manuscript.

## Conflict of Interest Statement

The author declares that the research was conducted in the absence of any commercial or financial relationships that could be construed as a potential conflict of interest.
